# DTI Profiles for Rapid Description of Cohorts at the Clinical-Research Interface

**DOI:** 10.3389/fmed.2018.00357

**Published:** 2019-01-10

**Authors:** Christine Lock, Janell Kwok, Sumeet Kumar, Azlina Ahmad-Annuar, Vairavan Narayanan, Adeline S. L. Ng, Yi Jayne Tan, Nagaendran Kandiah, Eng-King Tan, Zofia Czosnyka, Marek Czosnyka, John D. Pickard, Nicole C. Keong

**Affiliations:** ^1^Department of Neurosurgery, National Neuroscience Institute, Singapore, Singapore; ^2^Department of Neuroradiology, National Neuroscience Institute, Singapore, Singapore; ^3^Department of Biomedical Science, Faculty of Medicine, University of Malaya, Kuala Lumpur, Malaysia; ^4^Division of Neurosurgery, Department of Surgery, Faculty of Medicine, University of Malaya, Kuala Lumpur, Malaysia; ^5^Department of Neurology, National Neuroscience Institute, Singapore, Singapore; ^6^Duke-NUS Medical School, Singapore, Singapore; ^7^Neurosurgical Division, Department of Clinical Neurosciences, University of Cambridge, Cambridge, United Kingdom

**Keywords:** normal pressure hydrocephalus, complex, comorbidities, MRI, DTI

## Abstract

Normal pressure hydrocephalus (NPH) is a syndrome comprising gait disturbance, cognitive decline and urinary incontinence that is an unique model of reversible brain injury, but it presents as a challenging spectrum of disease cohorts. Diffusion Tensor Imaging (DTI), with its ability to interrogate structural white matter patterns at a microarchitectural level, is a potentially useful tool for the confirmation and characterization of disease cohorts at the clinical-research interface. However, obstacles to its widespread use involve the need for consistent DTI analysis and interpretation tools across collaborator sites. We present the use of DTI profiles, a simplistic methodology to interpret white matter injury patterns based on the morphology of diffusivity parameters. We examined 13 patients with complex NPH, i.e., patients with NPH and overlay from multiple comorbidities, including vascular risk burden and neurodegenerative disease, undergoing extended CSF drainage, clinical assessments, and multi-modal MR imaging. Following appropriate exclusions, we compared the morphology of DTI profiles in such complex NPH patients (*n* = 12, comprising 4 responders and 8 non-responders) to exemplar DTI profiles from a cohort of classic NPH patients (*n* = 16) demonstrating responsiveness of white matter injury to ventriculo-peritoneal shunting. In the cohort of complex NPH patients, mean age was 71.3 ± 7.6 years (10 males, 2 females) with a mean MMSE score of 21.1. There were 5 age-matched healthy controls, mean age was 73.4 ± 7.2 years (1 male, 4 females) and mean MMSE score was 26.8. In the exemplar cohort of classic NPH patients, mean age was 74.7 ± 5.9 years (10 males, 6 females) and mean MMSE score was 24.1. There were 9 age-matched healthy controls, mean age was 69.4 ± 9.7 years (4 males, 5 females) and mean MMSE score was 28.6. We found that, despite the challenges of acquiring DTI metrics from differing scanners across collaborator sites and NPH patients presenting as differing cohorts along the spectrum of disease, DTI profiles for responsiveness to interventions were comparable. Distinct DTI characteristics were demonstrated for complex NPH responders vs. non-responders. The morphology of DTI profiles for complex NPH responders mimicked DTI patterns found in predominantly shunt-responsive patients undergoing intervention for classic NPH. However, DTI profiles for complex NPH non-responders was suggestive of atrophy. Our findings suggest that it is possible to use DTI profiles to provide a methodology for rapid description of differing cohorts of disease at the clinical-research interface. By describing DTI measures morphologically, it was possible to consistently compare white matter injury patterns across international collaborator datasets.

## Introduction

NPH was first described in 1965 by Hakim and Adams as a condition of “symptomatic occult hydrocephalus with ‘normal' cerebrospinal fluid (CSF) pressures” (1, 2). It classically comprises of a triad of gait disturbance, cognitive decline and urinary incontinence associated with ventriculomegaly in the absence of persistently elevated intraventricular CSF pressures. The diagnostic challenge is that the clinical features of NPH are commonly found in functional decline from aging or other neurodegenerative conditions. It is therefore possible that “many patients with a potentially reversible condition are misdiagnosed as having Alzheimer's disease or vascular dementia and *vice versa”* (3, 4). Although NPH is an apparently rare condition accounting for an estimated 5% of dementias, it is more likely that its true incidence is underestimated, due to the confounding factors of multiple comorbidities in the elderly population (5). However, unlike other conditions within the dementia spectrum, features of the NPH syndrome may be reversed by the insertion of a CSF shunt.

There is published data demonstrating that the condition is reversible across differing populations worldwide. Recent neurology practice guidelines concluded that there was evidence for “96% chance subjective improvement and 83% chance improvement on timed walk test at 6 months” and that shunting was possibly effective in idiopathic NPH (6). Somewhat surprisingly, increasing age in NPH does not decrease the chance of shunting being successful. Few conditions in the elderly are known to demonstrate such levels of response to intervention. This should therefore elevate the importance of the study of NPH within aging research as an urgent priority (7).

NPH presents as a challenging collection of patient cohorts along a spectrum of disease. Neuropsychological profiling, gait/ balance measurements and CSF infusion studies may help predict which patients have the potential to improve with surgical intervention. However, such techniques require significant patient cooperation for meaningful testing to occur. This may not be possible for patients presenting at the late or complex part of the NPH spectrum who are less able to participate in active testing methods. In these types of NPH cohorts, supplementary imaging methods to confirm and characterize NPH features are of critical importance. These methods provide supporting information in evaluating the NPH component remediable to CSF diversion in order to balance the risks vs. benefits of surgical intervention in patients where multiple clinical confounders co-exist.

Yet, one of the major obstacles in the development of novel tools for the interpretation of NPH imaging findings is that the pathogenesis for classic NPH is still unknown. Published data have been contradictory across different imaging modalities [see Keong et al., 2016 (8) for a comprehensive review]. Studies have demonstrated congruence with different hypotheses involving structural changes, cerebral blood flow and CSF hydrodynamics (9). It is thought that biomechanical forces, such as tissue distortion caused by ventricular dilatation may result in CSF and interstitial fluid stasis. This causes an increase of interstitial fluid pressure, leading to reversal of fluid flow, which then results in the failure of drainage of neurotoxic compounds such as amyloid-β (10, 11). Studies have also demonstrated reduced periventricular blood flow and impaired cerebrovascular autoregulation in NPH, suggesting that watershed ischaemia in the deep white matter and/or leakiness of damaged vasculature may be the starting point for the process of accumulation of toxic waste products that results in the increase of interstitial fluid pressure (11, 12). Conversely, increased CSF stroke volume through the aqueduct has been found in the NPH population (13–15) despite normal CSF pressures. These processes, or a combination of them, may disrupt the cerebral mantle and the white matter tract connections serving the cortex in a spectrum of injury processes. It is possible that “some types of disruption may be more tolerable (i.e., more reversible) than others” (16). Imaging methodology that is able to simultaneously document differing injury patterns, such as axonal loss, compression, stretching and/or oedema would be greatly advantageous in understanding the cohorts presenting with NPH.

Diffusion Tensor Imaging (DTI) is a methodology that lends itself to the understanding of intricate structural changes at a microarchitectural level by using mathematical modeling of water diffusion properties. As the displacement of fluid in compartments is critical within the NPH spectrum, studies have shown that DTI has been able to demonstrate different patterns of white matter injury consistent with the symptomatology of the NPH disorder (16). DTI has also been found to differentiate NPH from other cohorts such as those with other types of ventriculomegaly, chronic hydrocephalus as well as Alzheimer's disease, Parkinson's disease, and other dementias (17–19). However, there are challenges in DTI acquisition and interpretation that prevent its more widespread uptake at the clinical-research interface. As DTI imaging is performed at different technical specifications across multiple scanners and sites, there is a lack of understanding of how to harmonize interpretation of DTI measures and so, derive knowledge of injury patterns from different cohorts of disease. DTI post-processing and analysis methods may also be dependent on availability of software tools and computing infrastructure. This confounds the efforts of interested collaborators to share common findings across international working groups and to discover new targets for intervention.

In this study, we present the use of a novel methodological tool for DTI interpretation that illustrates the ideal of the new praxis of translational medicine, in which a patient-centered approach to disease is promoted and prioritized. In such an ideal, NPH patients within their respective patient cohorts presenting to international collaborators would have access to the same DTI interpretation and understanding of their disease process, through an ability to share common knowledge of imaging markers for thresholds of reversible vs. irreversible brain injury. In order to overcome the challenges of applying DTI interpretation techniques across collaborator sites, new tools are needed to address our gaps of understanding. We present the use of a simplistic DTI interpretation methodology that leverages on existing capabilities at the clinical/research interface to convert DTI measures into a consistent morphological classification for more rapid comparisons of clinical cohorts across sites.

## Materials and Methods

The study comprised a prospective cohort of patients with complex NPH undergoing management at the National Neuroscience Institute (NNI), Singapore. The study protocol was approved by the local research ethics committee (CIRB 2016/2627). A cohort of healthy controls was recruited under a subsequent study (CIRB 2017/2854). Written informed consent was obtained from all participants or, in cases of dementia (MMSE <24/30), their legal representatives, for inclusion in the study.

### Subjects

Thirteen patients diagnosed with complex NPH undergoing the extended CSF drainage protocol were selected for the study from the NPH programme at the National Neuroscience Institute, Singapore between 2016 and 2017. Participants were recruited with a particular focus on the complex NPH subtype (further described below), and therefore presented with multiple comorbidities co-existing. Additionally, five age-matched healthy controls who were functionally independent and had no neurological conditions were recruited from the population. A comparator dataset of a cohort of 16 patients with classic NPH attending Addenbrooke's Hospital, Cambridge University Hospitals NHS Trust and nine age-matched healthy controls, served as the exemplar for the analysis and interpretation of DTI profiles. Details of patient characteristics, study protocol, and ethical approval for the exemplar dataset, who were studied pre- and 2 weeks post-operatively after successful shunting, have been previously published (16).

### Protocol for NPH Programme

Patients accepted for testing in the NPH protocol had clinical descriptions consistent with either probable or possible NPH, according to criteria in published guidelines (20). All patients demonstrated communicating hydrocephalus, with ventriculomegaly defined as an Evans' index (maximum width of frontal horns of the lateral ventricles divided by the transverse inner diameter of the skull) ≥0.30, or a Bicaudate index (minimum intercaudate distance divided by the brain width along the same line) ≥0.25. Patients with probable NPH had at least two out of three features of the NPH triad of gait disturbance, cognitive impairment, and urinary incontinence. Patients with possible NPH either had (a) incontinence and/or cognitive impairment in the absence of an observable gait or balance disturbance or (b) gait disturbance or dementia alone. Within the NPH programme, we termed patients amenable to standard testing and management according to international guidelines as having “classic NPH.” Typically, these patients demonstrated significantly positive responses to high-volume tap testing and were offered shunt insertion without further supplementary testing. However, patients who demonstrated low/ borderline positive results on tap testing or had comorbidities confounding the assessment of short-term responsiveness to CSF drainage were offered the extended CSF drainage protocol.

We also identified a separate subtype of NPH patients presenting with multiple comorbidities co-existing, in particular overlay from vascular risk burden and neurodegenerative diseases. These patients had clinical symptoms and signs consistent with probable/possible NPH according to international and Japanese guidelines (20–22), and had strong neuroradiological features supportive of the NPH diagnosis. However, due to overlay, testing their CSF responsiveness was difficult. We termed this subtype as “complex NPH.” Further management was required to identify and optimize other concurrent conditions before testing. “Where NPH features coexisted with other neurological, psychiatric, or general medical disorders, symptoms must be deemed not to be entirely attributable to these conditions” (20). In cases with neurodegenerative overlay, patients were referred for further evaluation via the NPH programme following confirmation that they did not fit diagnostic criteria for Alzheimer's and Parkinson's diseases, and/or had; limited response to disease-modifying drugs such as levodopa. Patients with cardiac risk were assessed as being of no higher than moderate risk for surgical intervention prior to being offered testing in the NPH programme.

All participants in this study had complex NPH and underwent insertion of a lumbar drain to facilitate the extended CSF drainage protocol. In two participants, failure of drainage led to the conversion of the lumbar drain to insertion of an Ommaya reservoir for testing. In one of the latter, significant psycho-behavioral issues resulted in failed MR imaging; this patient was subsequently excluded from the analysis. CSF drainage in this patient resulted in improvement in behavioral symptoms and the patient underwent completion of ventriculo-peritoneal shunting following their exit from the study.

The remaining 12 participants underwent the full NPH programme for CSF drainage, including clinical gait and cognitive testing, as well as pre- and post-drainage inpatient MR imaging. Patients with a lumbar drain *in-situ* underwent a 3-day drainage/7-day global assessment protocol, achieving ≥300 mls total CSF withdrawal whereas the patient undergoing serial reservoir taps had a modified protocol achieving ≥150–200 mls total CSF withdrawal to account for the tolerance needed for more rapid drainage and increased infection risk.

### Imaging Acquisition and Post-processing

All MR imaging data for this study were acquired with a 3.0-T MR scanner (Ingenia, Philips Medical Systems, Best, the Netherlands), including 3D T1, T2, FLAIR, and DTI sequences. DTI was obtained using a single-shot echo-planar sequence with a slice thickness of 2.3 mm. Images were acquired in 20 gradient directions with the following parameters: *b* = 0 and 1,000 s/mm^2^, TR = 7,274 ms; TE = 80 ms; FOV 220 × 220 mm; and matrix = 96 × 96, resulting in a voxel size of 2.3 × 2.3 × 2.3 mm, with SENSE factor of 2.5. A few patients were downgraded to the 1.5-T scanner at equivalent specifications due to MR safety concerns. All DTI processing was performed by using ExploreDTI (ExploreDTI, PROVIDI Lab, Utrecht, the Netherlands).

### DTI Analysis

Following corrections of subject motion and eddy current distortions, tract pathways were reconstructed using whole brain tractography. Due to dual technical constraints of scanning specifications and fiber distortion in the presence of significant ventriculomegaly, automated tractography extraction only reliably generated key periventricular white matter tracts. This “at-risk” model of white matter, including projection fibers (corticospinal tract), commissural/callosal fibers (corpus callosum, anterior commissure), and key association tracts (typically inferior longitudinal, fronto-occipital, and uncinate fasciculi) but excluding short association fibers, was found to be reproducible in all participants.

We performed DTI analysis and interpretation according to published methodology. We also derived disease-specific (*n* = 16) and human control exemplar data (*n* = 9) for DTI profiles from the published dataset (16). DTI measures, involving six similar regions-of-interest (ROI), were used to generate an overall estimate of the exemplar for mean DTI measures of periventricular white matter. We have previously confirmed the comparability and reliability of DTI measures, extracted using ROI methodology, across different preferred software tools (16).

### Gait, Balance, and Cognitive Assessments

Patients underwent physiotherapy-led examinations of the 10 m walking test, Tinetti gait and balance examination, and had a Mini-Mental State Examination (MMSE) carried out by occupational therapists. Inpatient assessment was further corroborated with the patient's own reported measures of functional performance at home in the early period following discharge. Using a simple report scale (from – to +100% levels), patients and/or caregivers were asked to grade their own perceived levels of improvement or deterioration at home to the nearest 10%, with 0 being no perceivable difference, following admission for CSF drainage. A positive response to CSF drainage was defined as an increase of ≥10% in any measure of inpatient gait, balance or cognitive testing (23) and ≥20% functional improvement on the patient's own self-report measure.

### Statistical Analyses

Analyses were performed using SPSS Statistics Version 23.0 (IBM Corp., Armonk, NY, USA). Between-group and within-group comparisons for DTI measures were tested with paired-samples and independent-samples *t*-test. Mann–Whitney U and Wilcoxon signed-rank tests were used for other variables. Spearman's rank correlation was used for correlations. All statistical tests were two-tailed and significance level was set at *p* < 0.05. All group means and DTI profile graphs were generated with Microsoft Excel Version 15.23 (Microsoft Corp., Redmond, WA, USA).

## Results

### Clinical Characteristics

Following one exclusion, the study cohort included 12 participants (10 males, 2 females) with mean age 71.3 ± 7.6 years. All patients presented with gait disturbance, 58.3% had cognitive impairment, and 58.3% had urinary incontinence or known bladder dysfunction (Table 1). All patients with complex NPH completed a baseline MMSE pre-drainage; mean MMSE was 21.1. MMSE scores were not significantly different between responder (Mean MMSE = 23.3) and non-responder (Mean MMSE = 20.0) groups. The cohort of complex NPH patients in the Singapore study was similar in clinical composition to the exemplar dataset derived from the Cambridge study of classic NPH patients (*n* = 16) in terms of gender (10 male, 6 female) and age (mean age of 74.7 ± 5.9 years), but differed in ethnicity. Classic NPH patients presented with mean MMSE = 24.1, just above the dementing range. The age-matched healthy controls in the Singapore study (1 male, 4 female) had a mean age of 73.4 ± 7.2 years and mean MMSE was 26.8. The Cambridge exemplar dataset had nine age-matched healthy controls (4 males, 5 females; mean age of 69.4 ± 9.7 years) and mean MMSE = 28.6, the best of all available cohorts.

**Table 1:** Clinical characteristics of complex NPH patients.

	**Age**	**Sex**	**MMSE**	**NPH symptoms**	**Other comorbidities**
NNPH01	77	M	16	Gait disturbance, memory impairment, urinary incontinence	Hypertension, diabetes mellitus, IHD, multifactorial dementia, CKD
NNPH03	74	M	26	Predominantly gait disturbance	Hypertension, parkinsonism
NNPH04	72	M	28	Predominantly gait disturbance	Parkinsonism, previous stroke
NNPH05	74	F	11	Gait disturbance, memory impairment, urinary incontinence	Hypertension, hyperlipidaemia, diabetes mellitus, vascular parkinsonism, dementia
NNPH06	73	F	15	Gait disturbance, memory impairment, urinary incontinence	Hypertension, hyperlipidaemia, diabetes mellitus, vascular dementia, bladder dysfunction
NNPH07	67	M	21	Gait disturbance, memory impairment, urinary incontinence	Hypertension, hyperlipidaemia, lumbar spondylosis, and degenerative disc disease
NNPH08	71	M	28	Predominantly gait disturbance, mild memory impairment, urinary frequency	Hypertension, hyperlipidaemia, diabetes mellitus, IHD, SIADH
NNPH09	81	M	29	Gait disturbance, urinary incontinence	Hypertension, hyperlipidaemia, parkinsonism, cervical spondylosis
NNPH10	67	M	20	Gait disturbance, memory impairment, urinary incontinence	Hypertension, hyperlipidaemia, diabetes mellitus, aortic sclerosis, cervical spondylosis
NNPH11	55	M	12	Gait disturbance, memory impairment, urinary incontinence	Hypertension, Korsakoff's syndrome, behavioral disturbance
NNPH12	82	M	17	Predominantly gait and cognitive disturbance	Hypertension, hyperlipidaemia, IHD, parkinsonism, COPD
NNPH13	63	M	30	Predominantly gait disturbance, urinary frequency	Hyperlipidaemia, cervical and lumbar spondylosis

*IHD, ischaemic heart disease; CKD, chronic kidney disease; SIADH, Syndrome of inappropriate antidiuretic hormone secretion; COPD, Chronic obstructive pulmonary disease*.

In the current study, eight out of the 12 patients were able to complete a 10 m walking test at 0, 48, and 72 h CSF drainage. One patient could not be assessed at 48 h and their response was assessed purely on the last measure. One patient missed their baseline assessment and two patients were not able to undergo any gait testing; their scores were excluded from gait analysis and their response assessed based on other domains and functional improvement, such as level of dependence for sit/stand transfers or balance. Median time for the 10 m walk was 12.9 s (IQR = 11.8–28.5 s) at 0 h and 14.8 s (IQR = 12.8–19.1 s) at 72 h CSF drainage.

### Comparisons Between DTI Parameters

As expected, the majority of the cohort of complex NPH patients were non-responders. Of the 12 participants who were included in the analysis, four responded to CSF drainage and were subsequently offered definitive surgical intervention in the form of ventriculo-peritoneal shunting. We confirmed that DTI measures (FA, MD, L1, and L2and3) were statistically different between cohorts [complex NPH vs. classic NPH patients (*p* < 0.001), healthy controls in Cambridge vs. Singapore (*p* ≤ 0.001)]. When patients were compared to healthy controls within the individual sites, nearly all DTI measures were significantly different between groups. Pre-drainage, both complex NPH responders and non-responders demonstrated significant differences in MD (axonal disruption) and L1 (stretch/compression) compared to healthy controls. However, there were only significant differences in L2and3 compared to healthy controls in the complex NPH non-responder group (Table 2), suggesting that the white matter microstructure in the complex NPH responders was better preserved (less stretch/oedema). Following CSF drainage, only the group of complex NPH responders demonstrated changes in DTI measures sufficient to cause a significant overall change in FA (Table 3).

**Table 2:** Difference in DTI measures between complex NPH and healthy controls.

**DTI measure**		**MR1**	***p*-value**	**MR2**	***p*-value**
FA	% difference between Complex NPH (all) vs. HC	0.225	*0.921*	−0.450	*0.881*
	% difference between Complex NPH responders vs. HC	2.928	*0.323*	−0.901	*0.820*
	% difference between Complex NPH non-responders vs. HC	−1.126	*0.716*	−0.225	*0.939*
MD	% difference between Complex NPH (all) vs. HC	14.461	***0.023***	12.815	***0.019***
	% difference between Complex NPH responders vs. HC	9.236	***0.049***	11.038	***0.035***
	% difference between Complex NPH non-responders vs. HC	17.079	***0.012***	13.704	***0.036***
L1	% difference between Complex NPH (all) vs. HC	14.272	***0.008***	12.236	***0.009***
	% difference between Complex NPH responders vs. HC	10.310	***0.015***	10.270	***0.019***
	% difference between Complex NPH non-responders vs. HC	16.261	***0.005***	13.210	***0.019***
L2and3	% difference between Complex NPH (all) vs. HC	15.070	***0.043***	13.803	***0.031***
	% difference between Complex NPH responders vs. HC	8.525	*0.107*	12.212	*0.050*
	% difference between Complex NPH non-responders vs. HC	18.334	***0.020***	14.599	*0.056*

*Italics indicate p-values; bold indicate significant p-values at a significance level of 0.05*.

**Table 3:** Pre-lumbar drain vs. post-lumbar drain DTI; mean (SD).

**DTI measure**	**Cohort**	**WBT Pre-LD**	**WBT Post-LD**	**% change**	***p*-value**
FA	NNI Complex NPH (all)	0.445 (0.021)	0.442 (0.023)	−0.674	*0.514*
	NNI Complex NPH responders	0.457 (0.011)	0.440 (0.019)	−3.720	***0.044***
	NNI Complex NPH non-responders	0.439 (0.023)	0.443 (0.026)	0.911	*0.450*
MD	NNI Complex NPH (all)	9.530 (0.993)	9.393 (0.828)	−1.438	*0.385*
	NNI Complex NPH responders	9.095 (0.404)	9.245 (0.515)	1.649	*0.363*
	NNI Complex NPH non-responders	9.748 (1.148)	9.467 (0.973)	−2.883	*0.210*
L1	NNI Complex NPH (all)	14.420 (1.233)	14.163 (1.056)	−1.782	*0.251*
	NNI Complex NPH responders	13.920 (0.529)	13.915 (0.613)	−0.036	*0.978*
	NNI Complex NPH non-responders	14.671 (1.434)	14.286 (1.240)	−2.624	*0.251*
L2and3	NNI Complex NPH (all)	7.086 (0.874)	7.008 (0.728)	−1.101	*0.555*
	NNI Complex NPH responders	6.683 (0.344)	6.910 (0.472)	−0.036	*0.179*
	NNI Complex NPH non-responders	7.287 (1.006)	7.057 (0.854)	−2.624	*0.187*

*Italics indicate p-values; bold indicate significant p-values at a significance level of 0.05*.

### DTI Profiles Across NPH Cohorts

**Figure 1:**
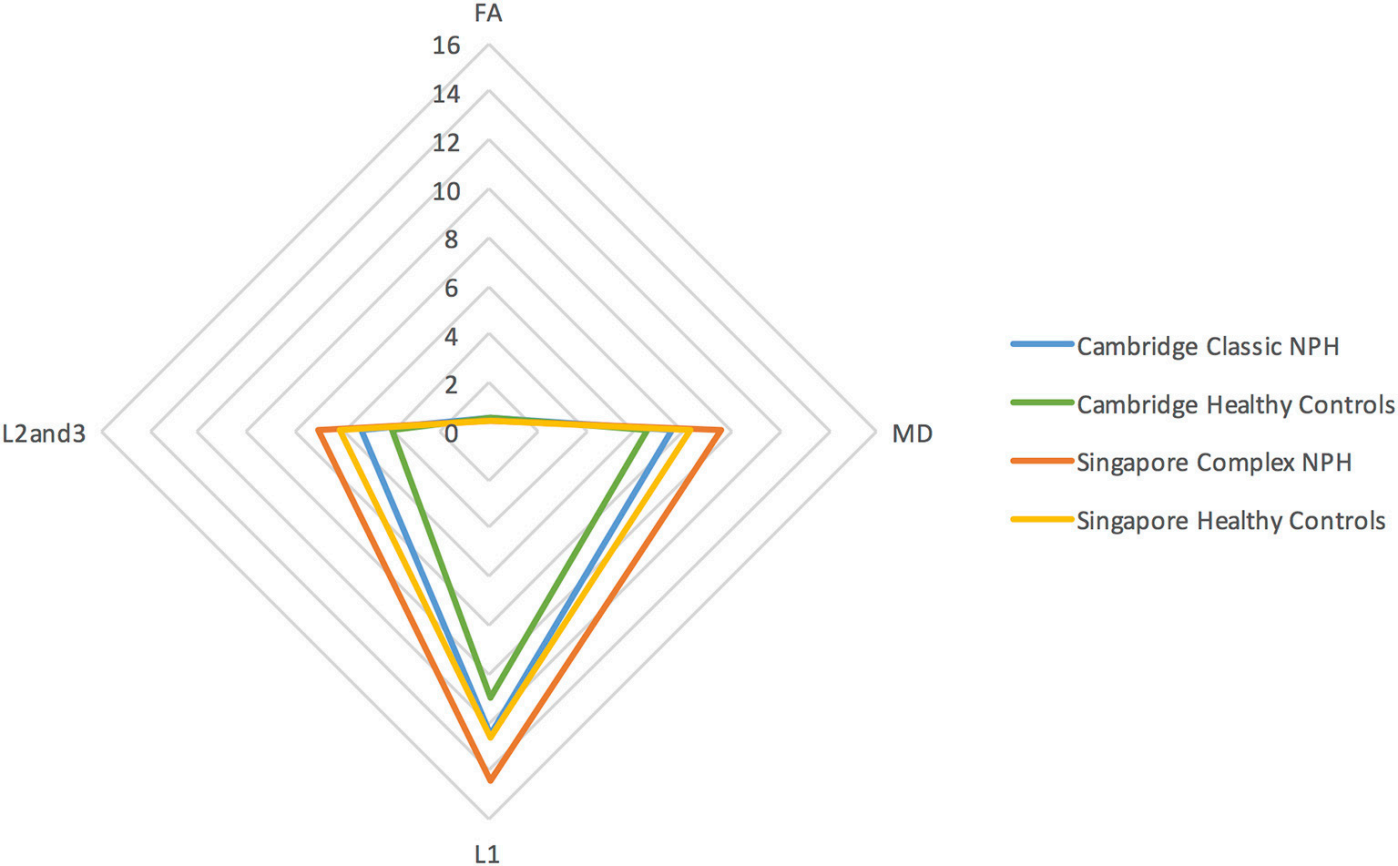
DTI profiles as radar graphs representing differences across classic NPH, complex NPH, and healthy control cohorts. Due to variations in scanning acquisition between collaborator sites, differences in DTI metrics may not be statistically meaningful. However, DTI profiles provide a methodological tool for comparability across cohorts. As NPH patients displayed worsening functional performance along the disease spectrum (for example, mean MMSE = 24.1 vs. 21.1 for classic vs. complex NPH, respectively), their DTI profiles concurrently worsened to match, across all diffusivity measures. The morphology of DTI profiles also matched the performance of healthy controls (mean MMSE = 28.6 vs. 26.8 for Cambridge vs. Singapore healthy controls).

**Figure 2:**
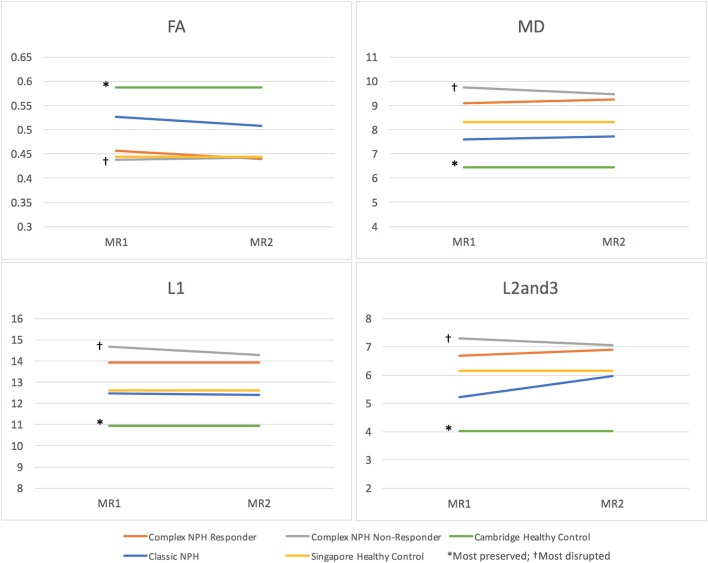
DTI profiles as line graphs across classic NPH, complex NPH (responder and non-responder), and healthy control cohorts. Differing NPH patient cohorts and healthy controls could be differentiated by the position of their DTI profiles within the spectrum of diffusivity measures. DTI profiles for Cambridge healthy controls^*^ were the most preserved (highest FA, lowest MD, L1, and L2and3), whereas DTI profiles for complex NPH non-responders^†^ were the most disrupted (lowest FA, highest MD, L1 and L2and3).

**Figure 3:**
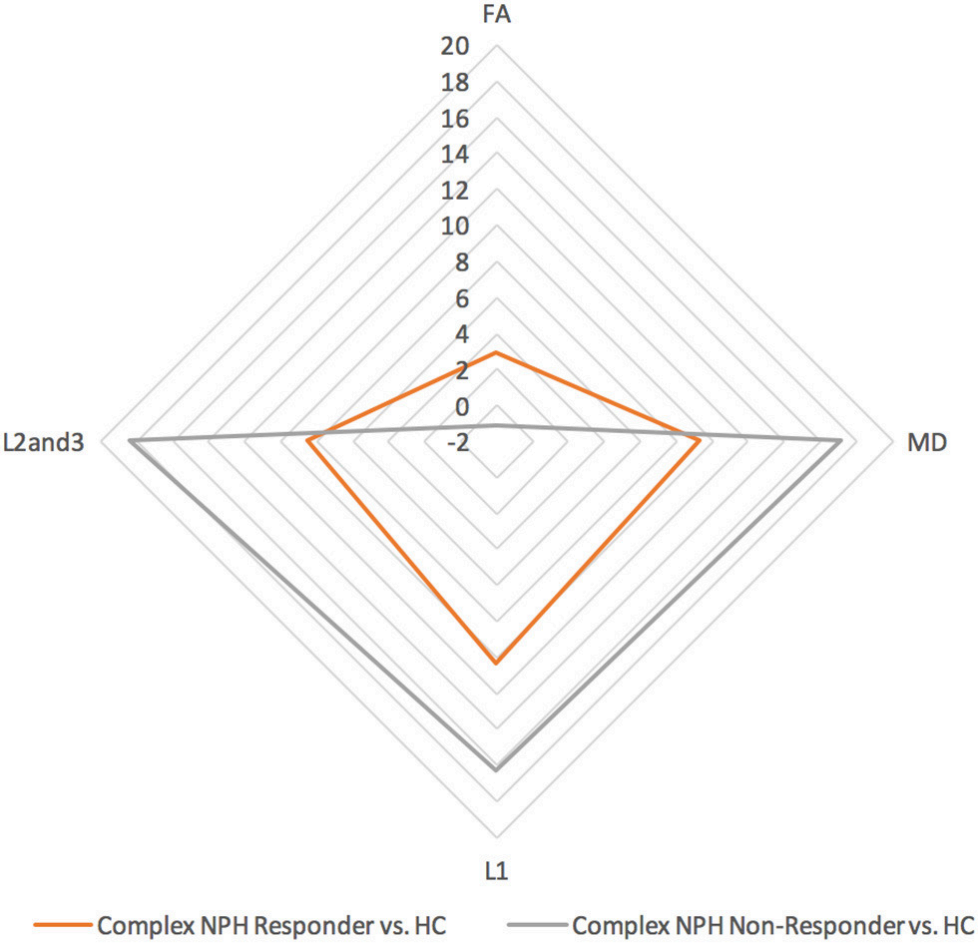
DTI profiles for complex NPH vs. healthy controls—radar graphs represent percentage (%) differences between pre-intervention patients and controls, differentiated by their post-intervention responses. The larger the DTI radar graph, the greater the differences between patients and controls. This graph demonstrates that compared to healthy controls, DTI profiles for complex NPH responders were more preserved compared to non-responders at baseline.

Differing NPH patient cohorts and healthy controls could be differentiated by the position of their DTI profiles within the spectrum of diffusivity measures (see Figures 1, 2). As NPH patients displayed worsening functional performance along the disease spectrum (for example, mean MMSE = 24.1 vs. 21.1 for Classic vs. Complex NPH, respectively), their DTI profiles concurrently worsened to match, across all diffusivity measures. The morphology of DTI profiles also matched the performance of healthy controls (mean MMSE = 28.6 vs. 26.8 for Cambridge vs. Singapore healthy controls). Nevertheless, in individual collaborator sites, DTI profiles for patients were consistently worse than controls across all diffusivity measures. When pre-intervention DTI profiles for complex NPH patients were plotted as percentage differences between patients and healthy controls (see Figure 3), DTI profiles for complex NPH responders were more preserved compared to non-responders at baseline.

### Morphology of DTI Profiles for Responders vs. Non-responders

**Figure 4:**
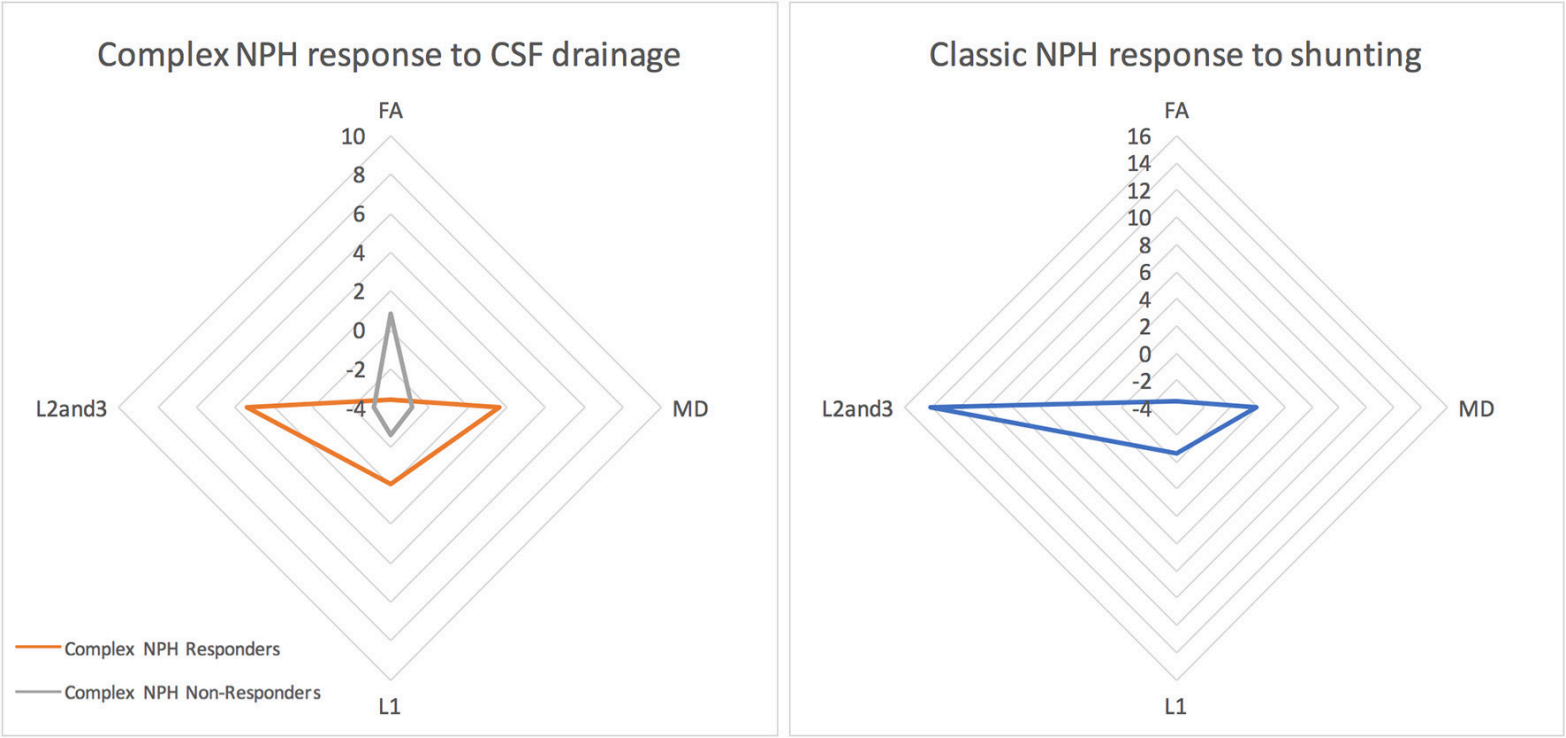
The morphology of DTI responses to CSF drainage—radar graphs represent percentage (%) changes between pre- and post-intervention DTI profiles in patients. The morphology of DTI profiles for complex NPH responders matched that of classic NPH responders, albeit with a differing magnitude of changes. DTI profiles for complex non-responders demonstrated entirely different morphology to responders with either complex or classic NPH.

**Figure 5:**
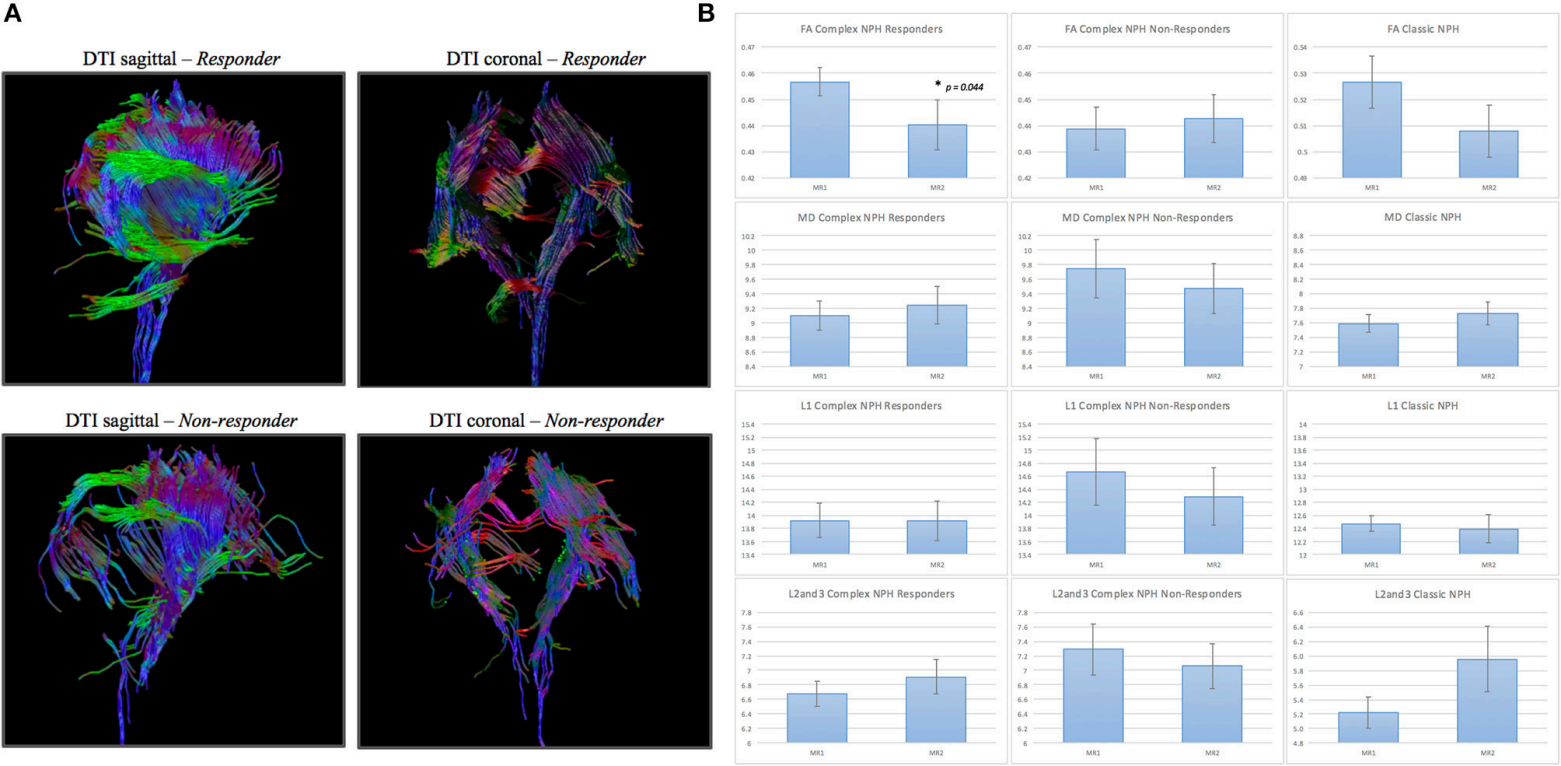
**(A)** DTI periventricular white matter tractography model. (Above, left) A typical complex NPH responder demonstrating structural integrity despite white matter disruption. (Below, left) A typical complex NPH non-responder demonstrating severe paucity of tracts and white matter damage. **(B)** DTI profiles (means with standard error bars) for complex NPH responders, non-responders and classic NPH cohorts. Patterns of directional changes in diffusivity measures for complex NPH responders were consistent with classic NPH, whereas directional changes for non-responders were suggestive of atrophy. MR1 = pre-intervention; MR2 = post-intervention.

**Figure 6:**
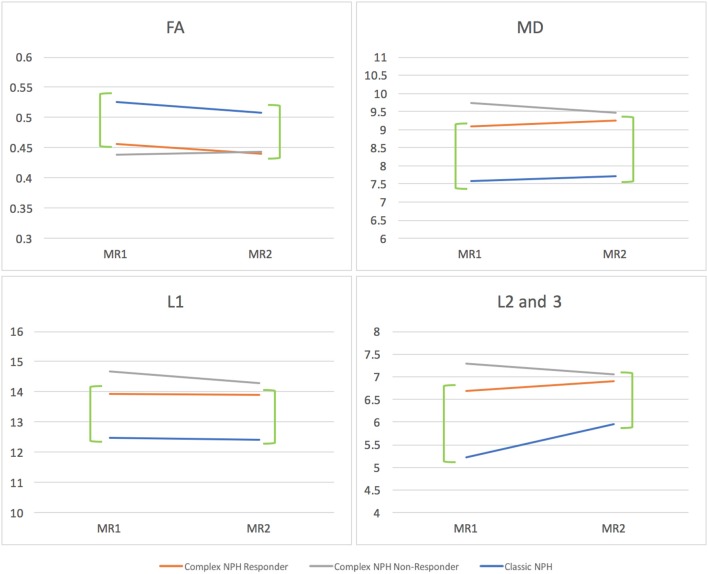
DTI profiles as line graphs comparing NPH cohorts across the spectrum (complex vs. classic NPH). DTI profiles for complex NPH responders mimicked patterns of changes seen in classic NPH patients across all diffusivity measures (green brackets). MR1 = pre-intervention; MR2 = post-intervention.

When the responses of NPH patients to CSF drainage were plotted as percentage changes between pre- and post-intervention diffusivity measures, the morphology of DTI profiles for complex NPH patients responding to CSF drainage matched that of classic NPH patients responding to successful shunting in the exemplar dataset, albeit with a differing magnitude of changes (see Figure 4). However, when the percentage changes pre- and post-intervention were plotted for complex NPH non-responders, DTI profiles demonstrated entirely different morphology compared to responders from either complex or classic NPH cohorts. When the means of diffusivity parameters were considered concurrently, changes in DTI profiles for complex NPH responders in Singapore undergoing extended CSF drainage mimicked patterns of changes seen in predominantly shunt-responsive patients with classic NPH in Cambridge. Such patterns [decreased fractional anisotropy (FA), increased mean diffusivity (MD), decreased axial (L1) with increased radial diffusivities (L2and3)] were seen consistently across all diffusivity measures (see Figures 5, 6). Furthermore, such patterns were not seen in complex NPH non-responders, who often exhibited changes in the exact opposite direction to responders. Instead, the changes seen in non-responders (an increase in fractional anisotropy (FA), with passive reduction of all other diffusivity measures following CSF drainage) were consistent with water diffusivity patterns in the presence of atrophy. The interpretation of diffusivity measures using DTI profiles also corresponded with visual representations of tractography models for responders vs. non-responders (Figure 5).

### Correlation of CSF Responsiveness From Lumbar Drainage With Surgical Outcome

Five patients (four responders and one patient excluded from analysis due to lack of imaging compliance) underwent surgical intervention, with six ventriculo-peritoneal shunts placed. All responders maintained their predicted responses following shunting. One responder developed a delayed abdominal pseudocyst with subacute infection. He had no evidence of infection on CSF sampling but had drainage of the pseudocyst with temporary shunt externalization, due to concerns of ascending infection. He subsequently had a shunt reinsertion on the contralateral side without neurological deterioration. Two responders reported post-operative improvement exceeding that of testing levels. At 1 year post-shunting, all complex and classic NPH responders maintained their good outcomes. Good outcomes were primarily reflected in improvement of gait symptoms. In terms of urinary symptoms, one responder had subjective improvement in incontinence and two responders reported no change; one responder had known pre-existing bladder dysfunction. One non-responder subsequently died from a cerebrovascular accident, a known comorbidity, outside the study. Another had delayed improvement at home but declined surgery.

## Discussion

In this study, we demonstrate the utility of using the morphology of DTI parameters to compare DTI profiles across collaborator sites, despite significantly different datasets. Due to the differing MR scanners and specifications unique to each collaborator site, it would not ordinarily be possible to directly compare DTI findings. The methodology of DTI profiles provides a framework for the rapid characterization of diffusivity measures to describe patient cohorts at the clinical-research interface that supports the new praxis of patient-centered international collaborative research.

### Technical Challenges of Data Harmonization

The challenges of comparing imaging datasets between differing collaborating units are well-accepted. Apart from MR field strengths, an analysis study by Cetin Karayumak et al. (24) illustrated differences in inter- and intra- specific scanners. For accurate analysis, harmonization and resolution of differences both across and within scanners are usually required. Inter-scanner differences can be found in both DTI measures such as fractional anisotropy and mean diffusivity, and structural measures, such as cortical thickness, and range from tissue-specific to regional differences. Types of intra-scanner differences include variability in receiver coils, reconstruction algorithms, magnetic fields, and acquisition parameters (25). Several methods have been developed to correct such issues. To combat structural variability, it is possible to use a physical phantom framework for monitoring and detection of scanner-related changes (26). Techniques such as generation of z-scores (27–29) and regression of covariates (30, 31) have been used to statistically resolve the differences in specific diffusion measures. These challenges can confound the establishment of international thresholds for comparison of cohorts within a spectrum of disease.

Whilst such work is still necessary, good scan-rescan repeatability and cross-scanner comparability have already been confirmed across differing sites and scanners, supporting the feasibility of using DTI measures derived from multiple collaborating sites (32). Ongoing efforts have focused upon achieving harmonizing of acquisition parameters and applying relevant corrections to the generation of DTI metrics. However, few studies have sought to understand the comparability of using the morphology of DTI changes as a form of consistent DTI interpretation across scanner sites. In this study, we present a simplistic methodology for use at the clinical-research interface that does not seek to directly address correction of diffusivity measures *per se*. Instead, we propose the use of DTI profiles to enable collaborators to confirm and characterize the comparability of their cohorts by providing a further layer of transparency, prior to the application of higher statistical methods. The checks afforded by DTI profiles also enable rapid detection of outliers for diffusivity parameters expected for local cohorts and allows individual units to ascertain if their presenting disease cohorts are comparable to international or open-access datasets of disease. The ability to perform these checks at the clinical-research interface contributes to the development of patient-centered collaborative research networks.

### NPH as a Model of Disease Cohorts

NPH as a disease continuum is critical to the development and study of DTI profiles because it serves as a both a model of reversible and irreversible brain injury. In our previous work in classic NPH patients, we have shown that it is possible to use DTI methodology to confirm and characterize differing pathophysiological processes occurring concurrently (16). Compared to age-matched healthy controls, NPH patients exhibited “distinct profiles of white matter injury.” These profiles could be entirely described by changes in anisotropic indices that are specific to the individual white matter tracts. We found that patterns of changes were influenced by measurable neuroanatomical factors and that some patterns of injury demonstrated a greater potential for reversibility than others (16). Predominant transependymal diffusion and stretch/oedema patterns found in the corpus callosum and inferior fronto-occipital/uncinate fasciculi, were less amenable to surgical intervention. By contrast, in white matter tracts where a pattern of stretch/compression was present, such as in the inferior longitudinal fasciculus, or where stretch/compression was the predominant pattern, such as in the posterior limb of the internal capsule, it was possible to demonstrate significant changes as early as 2 weeks following surgery (16). These changes, consistent with improvement, preceded changes in clinical outcome and were ultimately predictive of them. In this study, we have similarly demonstrated that certain patterns of DTI morphology are more responsive to CSF drainage than others. Complex NPH patients who did not respond to extended CSF drainage exhibited patterns consistent with axonal disruption and stretch/oedema (increased mean (MD), axial and radial (L1 and L2and3) diffusivities compared to healthy controls). Complex NPH responders demonstrated worse DTI measures that patients with classic NPH. However, white matter patterns in responders appeared to be more preserved, consistent with axonal disruption and stretch/compression [increased mean and axial diffusivities (MD and L1) but radial diffusivities (L2and3) not significantly different to healthy controls]. By plotting changes in DTI measures concurrently as profiles, it is possible to overcome the methodological challenges of comparing statistically significant datasets.

### DTI Profiles for the Rapid Comparison of Cohorts

Similarly, we have found, using DTI profiles, that despite confounding factors such as comorbidities involving cardiovascular risk burden and overlay from neurodegenerative disease, complex NPH responders can be shown to demonstrate patterns that mimic classic NPH responders across all diffusivity measures. The changes exhibited by non-responders are different and exhibit DTI morphology that are distinct from responders. DTI is useful because of its ability to characterize microarchitectural changes within white matter. The application of DTI profiles allows for the interpretation of contrasting pathological mechanisms without assumed prior knowledge of the predominant patterns of changes leading to its clinical syndrome. Our methodology of DTI profiles provides a mechanism for describing morphological changes common to different cohorts within the same spectrum of disease. This shorthand does not require that such groups are homogenous; we have demonstrated its utility within separate and significantly different patient cohorts within NPH, suggesting the DTI profiles for the disease process is proportionately more important than variations of the disease phenotype within its spectrum.

### Limitations

Our study has several methodological shortcomings. Firstly, our sample size is small, albeit relevant for the disease in question. The current study reflects improvements in DTI acquisition and analysis that is in some ways superior to that of the exemplar dataset. An optimal comparator study would involve simultaneous recruitment of different patient cohorts across both collaborator sites, and would require harmonization of DTI acquisition protocols in real time. Further validation work regarding the use of DTI profiles in comparison with international open-access datasets would be most helpful in this regard.

In conclusion, comparability of DTI measures across disease cohorts and collaborative sites is an obstacle to its usability and application at the clinical-research interface. Our findings suggest that it is possible to overcome such challenges by the use of DTI profiles to understand the morphology of microstructural changes and to apply such characterization in consistent terms to international collaborator datasets. The use of such methodology should be considered within the context of interdisciplinary collaboration as part of the new praxis of translational medicine.

## Ethics Statement

This study was carried out in accordance with the recommendations of the SingHealth Centralized Institutional Review Board (CIRB) with written informed consent from all subjects or their legal representatives, in the event that the subjects were not able to consent for themselves. All subjects gave written informed consent in accordance with the Declaration of Helsinki. The protocol was approved by the SingHealth Centralized Institutional Review Board.

## Author Contributions

NCK and CL contributed to the conceptualization and design of the study and wrote the manuscript. JK coordinated study procedures and scanning for the study. JK and CL contributed to the collection, analysis and interpretation of study data. NCK supervised study procedures and data collection for the study, and contributed to the analysis and interpretation of study data. SK contributed to design of imaging acquisition parameters and optimization of the study protocol as well as interpretation. NK, E-KT, and AN contributed to the clinical and research characterizations of the complex NPH patients. JP, MC, and ZC contributed to the exemplar dataset of the classic NPH patients. VN, AA-A, AN, and YJT contributed to the validation of the methodology of DTI characterization of the complex NPH patients using fluid markers (not presented in this manuscript). All co-authors contributed to revisions of the manuscript.

### Conflict of Interest Statement

The authors declare that the research was conducted in the absence of any commercial or financial relationships that could be construed as a potential conflict of interest.
